# Sliding Ferroelectric Catalyst for Carbon Nanoparticle Generation

**DOI:** 10.1021/acsanm.5c01032

**Published:** 2025-06-02

**Authors:** Johana Vaníčková, Klára Uhlířová, Jiří Volný, Tim Verhagen

**Affiliations:** † Institute of Physics, 86889 Czech Academy of Sciences, Na Slovance 2, Prague 8, 182 00, Czech Republic; ‡ Faculty of Mathematics and Physics, 138735 Charles University, Ke Karlovu 3, 121 16, Prague 2, Czech Republic

**Keywords:** 2D Materials, Moiré, Misfit Layer Compound, Catalysis, Sliding Ferroelectric, Carbon Nanoparticles

## Abstract

Two dimensional (2D) materials can be converted into sliding ferroelectrics when they are stacked on top of each other with a small twist angle between them. We show that the sliding ferroelectric surface is catalytically active at ambient, room-temperature conditions. The presence of ferroelectric domains with sizes up to tens of micrometers allows us to straightforwardly follow the conversion from hydrocarbons into carbon nanoparticles on moiré domains with the right polarization direction. The process can be significantly accelerated by using white light illumination. This catalytic reaction illustrates the huge potential 2D, twisted moiré materials can have in catalysis and surface chemistry control.

## Graphical abstract

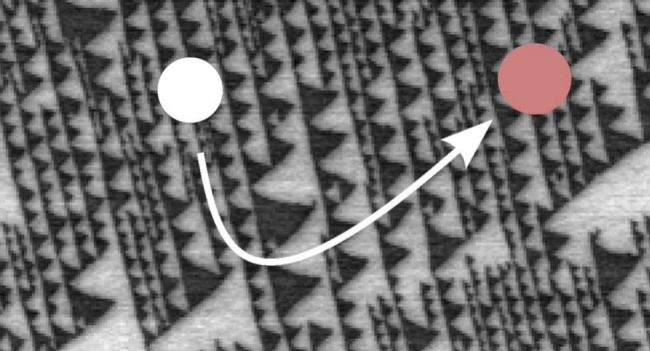

## Introduction

The properties of stacked two-dimensional (2D) materials can be significantly changed when the layers are stacked with a certain twist angle with respect to each other, such that a moiré landscape is created. It has been recently shown that 2D ferroelectric materials, so-called sliding ferroelectrics, can be straightforwardly created if two or more 2D layers are stacked in such a way that the inversion and/or mirror symmetry is broken.^1–8^

Due to its unusual physical properties, such as metallic ferroelectricity,^9^ ferroelectric nonlinear Hall effect,^10^ and the effortlessness to create a stable out-of-plane polarization down to bilayer thickness,^1–8^ sliding ferroelectricity has caught the attention of the scientific community. Sliding ferroelectricity is however not limited to bilayer structures, as each 2D layer in a multilayer stack can individually slide or twist, thereby generating a significant number of potential ways to stack the individual layers and induce a variation of possible polarization states.^2,3,11^

Although the physical properties of these van der Waals heterostructures are actively investigated, the link between surface chemistry, catalysis, and polar surfaces has barely been touched experimentally.^12–15^ The ability to control the twist between layered 2D materials introduces an extra control parameter to tune the properties of the material. For example, the twist allows the control of electron transport within the layer, but also at the solid–liquid interface.^13^ Furthermore, the twist angle can control the Gibbs free energy. Bilayer NbS_2_ with a twist angle of 5.08° is predicated to be close to the top of the volcano plot of the hydrogen evolution reaction, with even a smaller Gibbs free energy than Pt, the best electrocatalyst up to now.^14^ In addition, the bandgap can be locally modified due to the twisting of the layers, such that the light absorption and carrier dynamics of the photogenerated carriers can be locally controlled, as shown recently in a BiOCl moiré superlattice.^16^ A clear hint of the outstanding catalytic properties of stacked layers with a broken symmetry can be found in rhombohedral stacked (3R) transition metal dichalcogenides (TMDs) MoS_2_,^17^ WS_2_,^17^ TaS_2_,^18^ and NbS_2_.^19^

The potential of the ferroelectric surface has been known since the 1950s when Parravano^20^ found anomalies in the catalytic oxidation rates of CO over NaNbO_3_ and KNbO_3_ for temperatures below and above the ferroelectric Curie temperature. The selective absorption/desorption on polarized domains with a different polarization direction and the ability to separate electrons and holes to enhance the catalytic efficiency makes catalysis based on ferroelectrics a promising field. 2D ferroelectrics offer the additional advantage that the surface-to-volume ratio can be maximized, such that an “all surface reaction” can be achieved.^21^

At the boundary of a ferroelectric material, the spontaneous ferroelectric polarization induces a surface charge, which can be compensated by free charge carriers or defects in the bulk material or by adsorbed charged molecules. In ambient conditions, the presence of a thin water or hydrocarbon film plays an important role in the stabilization of the out-of-plane ferroelectric polarization via screening of the depolarization field.^22^ In 2D materials, it is well-known that on a freshly cleaved surface within an hour an unordered contamination layer consisting of water and diverse hydrocarbons grows. After exposing the surface layer for several days at ambient conditions, the unordered layer converts slowly into a self-organized molecular layer consisting of alkanes with lengths of 20 to 26 carbon atoms.^23,24^

In ferroelectric materials, spontaneous polarization can be controlled using diverse external stimuli such as a variation in the temperature, light illumination, or an external electrical field. A variation in the sample temperature will change the permanent spontaneous polarization, the pyroelectric effect, and thereby also the surface charge density.^25^ The pyroelectricity-generated positive and negative charges at the surface can participate in a redox reaction at the surface. Such pyrocatalytic reaction has been recently shown to happen when thermal cycling ferroelectric nanoparticles, for example, to degrade Rhodamine B dye,^26,27^ generate H_2_,^27^ reduce CO_2_,^28^ or create reactive oxygen species.^29^ Similar catalytic reactions triggered by mechanical vibrations, the piezoelectric effect, can be observed in BiFeO_3_ nanoparticles,^30,31^ or light can also control catalytic reactions in ferroelectric materials.^32^

Recently, we have shown that in the misfit layer compound (MLC) (PbS)_1.11_VS_2_, sliding ferroelectric moiré domains of tens of micrometers can be created.^33^ MLCs are a member of the TMD class of materials, with the general formula (MX)_1+*m*_(TX_2_)_*n*_, where M = Sn, Pb, Sb, Bi, or a rare earth element; T = Ti, V, Cr, Nb, or Ta; X = S or Se; 0.08 < *m* < 0.28; and *n* = 1, 2, or 3. As can be seen in the transmission electron microscopy (TEM) image in Figure 1d, the structure of the MLC consists of alternated stacked TMD and post-transition metal chalcogenide (TMM) monolayers.^34^ Both the TMM and TMD layer can be indexed using a monoclinic unit cell, and the *a* and *c* parameters of both systems have the same length: PbS has unit cell parameters *a* = 5.7062(5), *b* = 5.7674(7), *c* = 23.691(2) Å, and β = 94.878(8)°, and VS_2_ has *b* = 3.204 Å.^33^

**Figure 1:**
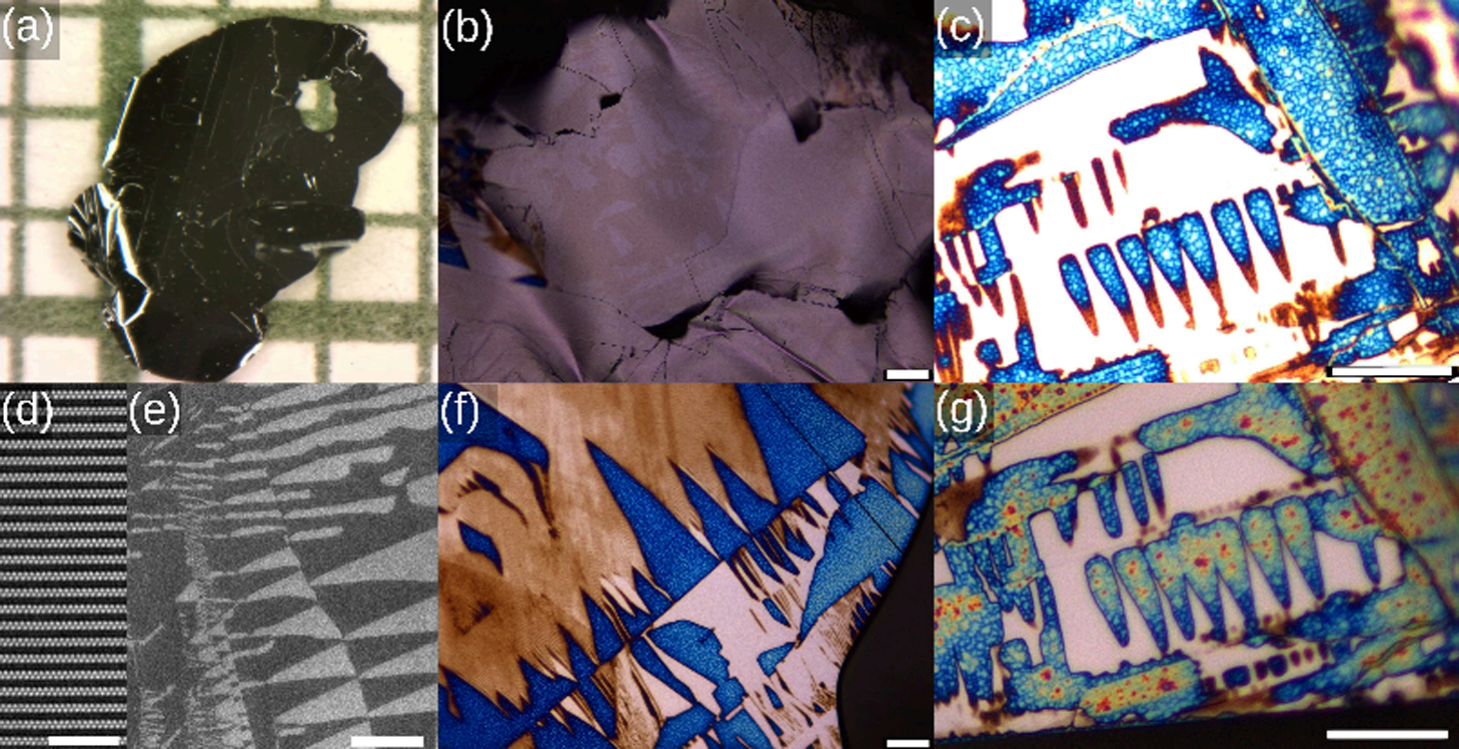
Microscopy images of the surface of (PbS)_1.11_VS_2_ crystals. (a) Optical microscopy image of a (PbS)_1.11_VS_2_ crystal on millimeter paper. (d) High-angle annular dark-field scanning transmission electron microscopy cross section of the crystal showing the layered structure. The Z-contrast image shows gray PbS and black VS_2_ layers. (e) Secondary electron microscopy image of a (PbS)_1.11_VS_2_ crystal, cleaved under ambient conditions with a complex moiré domain structure. The initial appearance of light-brown domains with carbon nanoparticles is visible in panel b. The light-brown domains subsequently evolve to darker-brown areas and subsequently light-blue and dark-blue regions, as visible in panel f. Panels c and g show the time evolution of the same area. The optical image in panel g is taken two months after the image in panel c, showing a clear change in the domain landscape. The scale bar in panels b and f is 50 μm, in panel d 5 nm, in panel e 100 μm, and in panels c and g 25 μm. The crystals are slightly bent, resulting in slightly defocused areas in the image.

Here, we show that the hydrocarbon present at the ferroelectric moiré domains on the MLC (PbS)_1.11_VS_2_ can be converted to carbon nanoparticles with sizes varying from a few nanometers up to hundreds of nanometers. Using a combination of optical microscopy, scanning probe microscopy, X-ray photoelectron spectroscopy (XPS), and Raman spectroscopy, the conversion from hydrocarbons to carbon nanoparticles is studied. The process can be significantly accelerated using white light illumination.

## Experimental Details

(PbS)_1.11_VS_2_ crystals were grown using chemical vapor transport according to a modified procedure of Gotoh et al.^33,35^ The pure elements (99.5% vanadium powder, small pieces of 99.999% lead, and 99.999% sulfur, all Alfa Aesar) with molar composition VPb_1.11_S_3.12_ were weighed and loaded in an approximately 25 cm long fused silica tube in an Ar-filled glovebox and subsequently sealed in vacuum of 10^–6^ mbar. The sample was first slowly heated in a vertical position up to 993 K and then placed in a two-zone horizontal furnace. The growth temperatures were 993 and 923 K in the hot and cold zone, respectively. The growth process took 1–6 weeks, depending on the desired size of the grown single crystals.

(BiS)_1.24_CrS_2_ crystals were grown using chemical vapor transport according to a modified procedure of Lafond et al.^36^ The pure elements (99.5% chromium powder, small pieces of 99.999% bismuth, and 99.999% sulfur, all Alfa Aesar) with molar composition CrBi_1.24_S_3.24_ were weighed and loaded in an approximately 25 cm long fused silica tube in an Ar-filled glovebox and subsequently sealed in vacuum of 10^–6^ mbar. The sample was first slowly heated in a vertical position up to 993 K and then placed into a two-zone horizontal furnace. The growth temperatures were 1023 and 963 K in the hot and cold zone, respectively. The growth process took 5 weeks.

Before the measurements, the crystals were placed using double-sided carbon tape on a Si substrate or metal disk and subsequently cleaved under ambient conditions. After cleavage, the samples were typically stored in polystyrene sample boxes or glass Petri dishes, either in a closed cabinet or on a lab bench. Scanning probe microscopy was conducted on an Asylum Research (Oxford Instruments, Goleta, CA, USA) Cypher S atomic force microscopy (AFM) instrument with an air temperature controller (Cypher ATC) using FESP AFM probes. Raman scattering measurements were carried out using a Renishaw Raman microscope (System 1000) with a 514 nm argon laser in the backscattering geometry. The laser fluency was adjusted such that during the long measurement the carbon layer was not visibly damaged by the creation of a black spot. Typical fluency used was 100 μW/μm^2^. Integration times on the order of 1 min with five accumulations were typically needed with this fluency. Raman data was fitted using in-house written Python script using LMFIT^37^ and Pandas.^38^

The optical response of the carbon particles on the bulk crystals was measured using a Thorlabs CCS200/M compact CCD spectrometer coupled with a fiber with a core diameter of 200 μm to a Leica DM2700 M optical microscope with white LED illumination (Leica LH113). The area probed with the optical fiber corresponds to approximately 4 μm with a 50×-magnification objective. The reflectivity of a clean area is scaled to the optical reflectivity measured using an ellipsometer.

The optical reflectivity of the bulk samples was measured by a Woollam RC2 ellipsometer in the spectral range from 0.74 to 6.4 eV using focusing probes, which decreases the light beam to a spot size of approximately 200 μm. The reflectivity is shown in Figure S3 in the Supporting Information.

The growth of the particles was studied in two regimes: natural growth and accelerated growth. During natural growth, the samples, placed in their sample boxes, were kept in ambient conditions for a certain time (time scale is weeks to months), whereas during the accelerated growth, white light, from a laser-driven light source (Energetiq Technology inc., EQ-99X-QZ-S) was focused on the sample using parabolic mirrors for a certain time (time scale is hours). The reflected light from the sample’s surface was collected each 5 s using a pair of parabolic mirrors and focused into an optical fiber connected to a Thorlabs CCS200/M compact CCD spectrometer.

Using a gallium-focused ion beam (FIB) scanning electron microscope FEI Helios NanoLab 660, a lamella was milled out perpendicular to the crystal surface, i.e., parallel with the *c*-axis. Scanning transmission electron microscopy images were acquired using an FEI Titan Themis cubed TEM operated at 300 kV.

XPS was measured on a K-Alpha instrument (Thermo Fisher Scientific Inc.) using monochromated Al Kα radiation and a spot size between 30 and 400 μm. Charging was compensated for using a flood source. The measured spectra were analyzed by using CASAXPS software. The core level spectra are fitted by using standard Voigt components with a Shirley background. During fitting, the number of Voigt components was kept as low as possible. The binding energy has not been shifted to match the C 1s core level to a “standard reference” value for adventitious carbon contamination.

## Results and Discussion

### Natural Growth of the Particles

(PbS)_1.11_VS_2_ crystallizes as a layer composite structure. In Figure 1a, an optical microscope image of a typical (PbS)_1.11_VS_2_ crystal on millimeter paper is shown. The crystals are typically a few mm^2^, and their thickness is of the order of a few tens of μm.

The ferroelectric domains in the crystals can be straightforwardly imaged using the secondary electrons (SE) in a scanning electron microscope.^39,40^ Figure 1e shows the typical domains observed in the (PbS)_1.11_VS_2_ crystals. A complex domain landscape is present, where the shape of the domains are preferentially aligned stripes or triangle-like.

In a typical time scale of weeks to months after cleaving the crystals, colored areas appeared on the surface, which changed color gradually, as can be seen in Figure 1b,c,f,g. First, regularly shaped areas become light brown, then gradually turn into dark brown and subsequently, dark blue, light blue, yellow and red. The regular-shaped areas, which change color, have shapes similar to the moiré domains observed in the SEM image in Figure 1e.

Besides changing color, the colored regions slowly grow in time, as can be clearly seen in Figure 1c,g, where the uncovered white triangles decrease slowly in dimension, whereas the blue region surrounding the triangles slowly grows in size. The difference between the images in Figure 1c,g is two months, indicating that the process of natural particle formation is relatively slow. Even after one year, still uncovered areas are visible, as can be seen in Figure S1 in the Supporting Information. The presence of uncovered areas after one year furthermore highlights that the crystals are stable for at least one year and do not degrade due to the ongoing surface reactions.

Using AFM, the different colored regions are studied in more detail. At the very first phase of particle creation, as can be seen in the AFM topography image in Figure 2a, particles with a diameter of just a few nanometers are present. Furthermore, they are only present on part of the ferroelectric moiré domains, which suggests that the process depends on the domain’s polarization direction. These small particles are barely visible in the optical images. Gradually, the particles grow up to dimensions of tens of nanometers.

**Figure 2:**
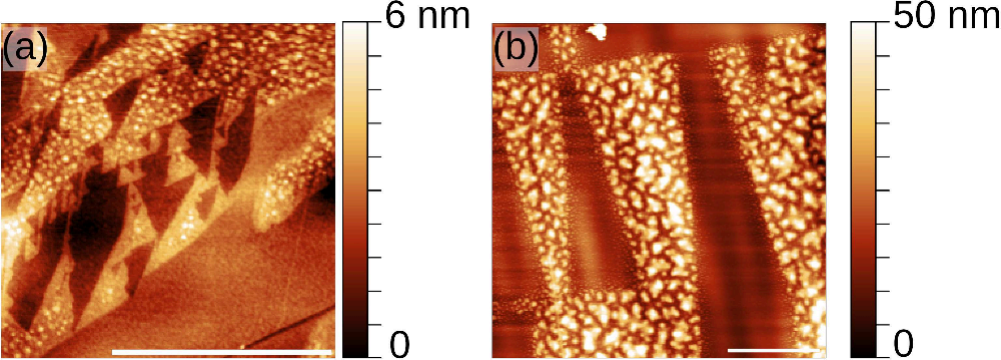
AFM topography of the surface of (PbS)_1.11_VS_2_ crystals. In panel a, the early phase of the particle formation is visible. Particles start to form on the lighter moiré domains, whereas on the darker regions, no particles are visible. In panel b, a different domain is shown, where larger particles are already grown. Within the triangular region, the growth of small particles is already visible. The scale bar in panels a and b is 4 μm.

In Figure 2b, an AFM topography image of a triangular region can be seen. In the center of the triangular area, no particles are visible, and toward the edge of the triangular domain, a gradual increase in particle size can be observed. As shown before by Hsieh et al.,^41^ particles deposited on moiré domains are able to locally change the domain configuration. Here, the particles on the edge of a domain move the domain boundary, thereby creating a fresh area for the growth of new particles. Outside the domain, large particle clusters are present.

By AFM, the typical size of the particles of several colored areas was determined. In light brown areas, the particles are approximately 10 nm (see Figure 2a), and in dark brown regions they have a size of 50 nm (see Figure 2b). The particles keep growing, and blue particles can easily reach 100 nm. When the particle size becomes larger, the particles slowly form a continuous film which hinders extraction of the particle diameter using AFM.

The optical reflectivity of the different colored areas was investigated with μ-reflection. Figure 3a shows the reflectivity of a clean area and areas with different colors. When particles start to grow and the sample slowly becomes brown, the reflectivity drops rapidly in the whole visible range. Subsequently, when the sample turns blue, the reflectivity increases around 400 nm, and when the sample becomes red, the reflectivity drops again in the 400 nm wavelength region and increases from 500 nm.

**Figure 3:**
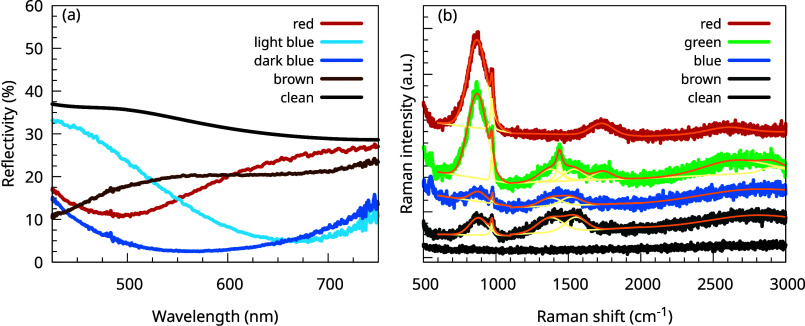
(a) Reflectivity and (b) Raman spectra of the clean (PbS)_1.11_VS_2_ crystal and of the (PbS)_1.11_VS_2_ crystal with carbon nanoparticles with different sizes, characterized by their color. The fitted Raman bands are shown using yellow, and the sum of the fitted bands in the D, G, and 2D regions is shown using orange. Each Raman spectrum is vertically offset for clarity.

The composition of the particles was explored by using Raman spectroscopy. In Figure 3b, typical Raman spectra of the samples are shown. A typical Raman spectrum of the clean (PbS)_1.11_VS_2_ crystal has only low-frequency Raman modes below 500 cm^–1^ (see Figure S2 in the Supporting Information).

Subsequently, Raman spectra are measured in the different colored areas. Although the Raman intensity of the thin layer is very weak, there is significant change of the Raman spectra above 500 cm^–1^, with broad peaks present around 1350, 1600, and around 2800 cm^–1^. Although the optical reflectivity changes significantly during the particle growth in the initial phase, as also can be seen in Figure 5, the Raman spectra do not change so dramatically. The changes of the Raman spectrum between the brown and blue particles is small, and therefore, only a spectrum for blue particles is shown.

As alkane chains are typically present on the surface of exfoliated 2D crystals,^23,24^ as a first attempt, the observed Raman modes will be correlated with the D, G, 2D Raman modes, which are typically present in disordered, amorphous, and diamond-like carbon.^42^

From the fitted D, G, and 2D Raman modes, information about the phase, disorder and the amount of hydrogen present in disordered, amorphous, and diamond-like carbon can be derived.^42^ Therefore, pseudo-Voigt peak shapes were fitted to extract the intensity, Raman shift, and full width at half-maximum of the different modes. The spectra were fitted with a minimum number of modes, and the fitted Raman modes are plotted together with the measured Raman data in Figure 3b. For the dark brown sample, Raman modes were found at 879, 972, 1374, 1550, and 2804 cm^–1^; for the blue sample at 878, 976, 1374, 1519, and 2738 cm^–1^; for the green sample at 871, 971, 1374, 1438, 1538, 1735, 2650, and 2908 cm^–1^; and for the red sample at 869, 972, 1730, and 2599 cm^–1^.

As can be seen, the Raman shift of the “G”-mode of the red spectra is located at 1730 cm^–1^, similar to one Raman mode in the green spectrum, whose value is well above the typical Raman shift for a G-mode in carbon. Furthermore, the intense modes between 800 and 1000 cm^–1^ are typically not present in disordered, amorphous, and diamond-like carbon.^43^

Therefore, to assign the Raman modes of the observed nanoparticles, a more careful inspection of the possible Raman modes in carbon-based materials is needed.^44–46^ As the starting materials are most probably alkane chains, in general, the presence of the modes between 800 and 900 cm^–1^ can be attributed to either C–C-stretching due to the terminal C–C­(=O)-group, CH_3_ rocking, or C–O stretching and the mode around 970 cm^–1^ to the =C–H deformation vibration.

In carbon-based materials, the Raman modes between 1350 and 1380 cm^–1^ can be typically attributed to CH_2_ wagging, the Raman modes between 1400 and 1500 cm^–1^ to CH_2_ or CH_3_ bending, the Raman modes around 1439 cm^–1^ to CH_2_ scissoring, the Raman mode between 1700 and 1750 cm^–1^ corresponds to C=O stretching, and the Raman modes in the region between 2800 until 3000 cm^–1^ to C–H stretching. Variation in the Raman shift and intensity can be caused by a variety of factors, such as the number of carbon atoms in the chain, the number of C=C bonds, respectively C=O or C–O bonds, the local stacking of the particles, and whether the nanoparticle is in the solid or liquid phase.

Typical catalytic reactions consist of several reaction steps, with adsorption and desorption of the reactants (here: hydrocarbons and water), intermediates, and the final product (here: carbon nanoparticles). In the presented case, reactions with unknown hydrocarbons present in the air take place; the full reactions that take place at the sample are therefore not known. Several possible reactions can however be identified, if we assume both alkane chains and water are present at the sliding ferroelectric domains, as shown in Scheme 1. It is known for ferroelectrics that at the negatively charged domains, the “contaminants” (H_2_O and hydrocarbons) can be reduced to anions (O^–^, OH^–^, COOH^–^, ...), whereas adsorption of the “contaminants” with a polar head (R–OH, R–COOH, ...) can happen at the positively charged domains.^22^ AFM microscopy does not show signs of degradation of the clean surface between the particles; therefore, the anions most likely react with the adsorbed molecules on the positively charged domains.

**Scheme 1:**
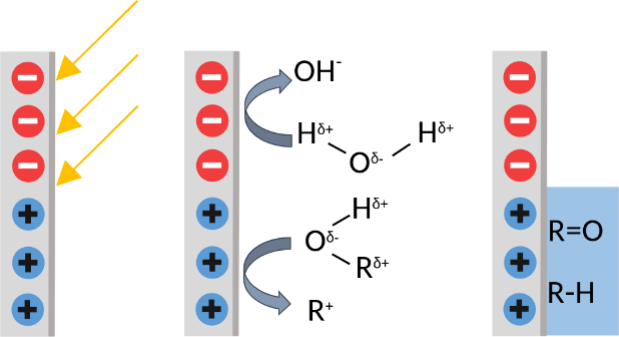
Schematic Diagram of the Proposed Reaction Mechanism to Grow Carbon Nanoparticles on a Sliding Ferroelectric Surface

The presence and increase of the Raman mode around 970 cm^–1^ hint that dehydrogenation of the alkane chains takes place. It should be mentioned that in the 890–1035 cm^–1^ region, also the V=O stretching vibration can be found.^47^ Oxidation of the VS_2_-layer might happen, but it should be noted that the observed Raman signal is most probably not caused by the small amount of oxidized VS_2_ compared to the tens of nanometers thick carbon particles. The crystal itself seems stable for more than 1 year, as can be seen from the different optical and AFM images. The presence of possible strong oxidants such as OH^–^ does not seem to visibly damage the crystal surface or the adjacent oppositely oriented domains. Second, for longer reaction times, the Raman mode between 1700 and 1750 cm^–1^ indicates that a carbonyl group is created.

To verify the Raman analysis, XPS spectra (see Figure 4) were obtained of three (PbS)_1.11_VS_2_ crystals with carbon nanoparticles grown on them using light illumination (see next section). As XPS is a surface-sensitive technique, only the content of the grown carbon nanoparticles should be reflected in the present spectra. This is the case, as the contribution of Pb corresponds only to approximately a few percent and V even less than one percent in the three measured samples. The main contribution is, as expected, carbon and oxygen.

**Figure 4:**
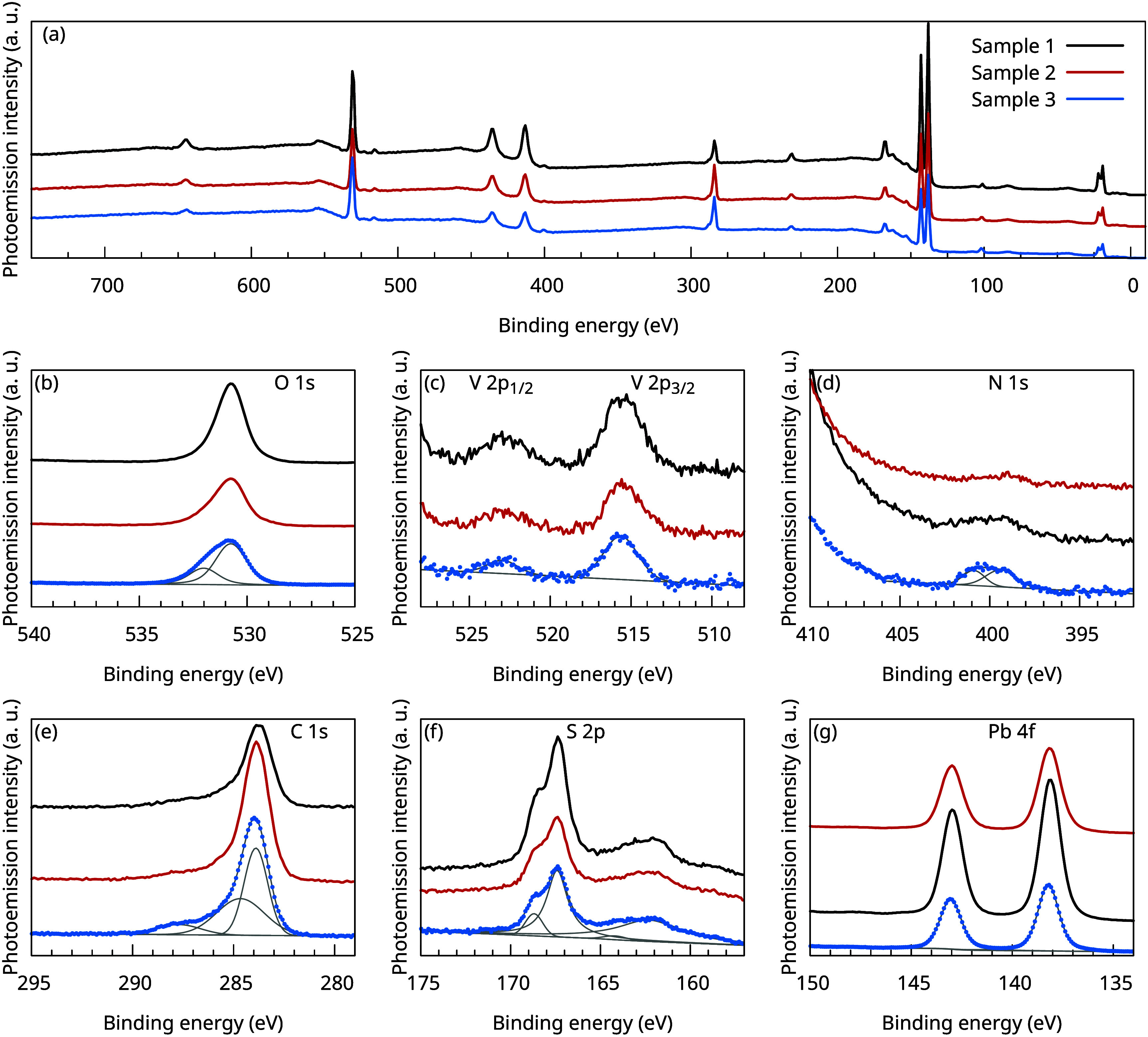
(a) XPS survey scan of the (PbS)_1.11_VS_2_ crystal with carbon nanoparticles on top. Panels b–g show O 1s, V 2p, N 1s, C 1s, S 2p, and Pb 5d core levels in detail, together with the for sample 3 fitted peaks and background.

Due to the complexity of the inhomogeneously grown carbon nanoparticles, which may contain a variety of particle sizes, defects, and functional groups, we will only mention a possible way to interpret the core level shape and position, without pretense of presenting an overall model.

The binding energy of the C 1*s* core level is centered at 283.8 eV, and in the shoulder, two more Voigt profiles centered at 284.7 and 287.7 eV can be fitted. As typically done for samples with adventitious carbon contamination, the C 1*s* centered core level at 283.8 eV could be shifted to 284.8 eV to compensate for the charging of the sample. However, here the carbon is grown on the sample and not adventitiously deposited. More reasons to not shift the binding energy can be found in the AFM images of particles and the optical reflection. The AFM images suggest that the particles have a three-dimensional sphere-like shape. Furthermore, the rich variety of colors and the change of the color of the particles during growth, as shown in Figure 3a, hint at a similarity with fullerene particles.^48^ Therefore, the C 1*s* core level centered at 283.8 eV might be linked to the C=C bond in pentagons.^49,50^ The fitted Voigt profile at the binding energy of 284.7 eV would than correspond to a “normal” C=C bond and the fitted Voigt profile at 287.7 eV to a C=O bond.

Furthermore, the presence of a broad core level at 515.6 eV, as shown in Figure 4c, can be assigned to a small amount of oxidized vanadium, in line with refs 33 and 51 . Similarly, the core levels of Pb 4*f* at 138.2 eV, as shown in Figure 4g, can be assigned to oxidized lead or oxidized lead sulfide.^52^

The natural growth of the particles indicates several things. First, as long alkane chains are present, the particles formed at the crystals are the leftover products of the principle reaction, as the evaporation rate of such long chains is low. Second, the continuous growth of the particles indicates that new carbon material in the air is captured during the reaction by the substrate and contributes to the reactions.

### Accelerated Growth of the Particles

To verify the reproducibility and as an attempt to improve the reaction speed, freshly cleaved crystals were illuminated by using focused white light. As can be seen in Figure 5a, already after several hours the color of the area in the elliptical light spot changed to various shades of brown. The variation in colors is caused by the presence of a wide variety of twin configurations, as visible in the SEM image in Figure 1d, which can have a different polarization state and reactivity due to the different twist angles of the stacked 2D layers.

**Figure 5:**
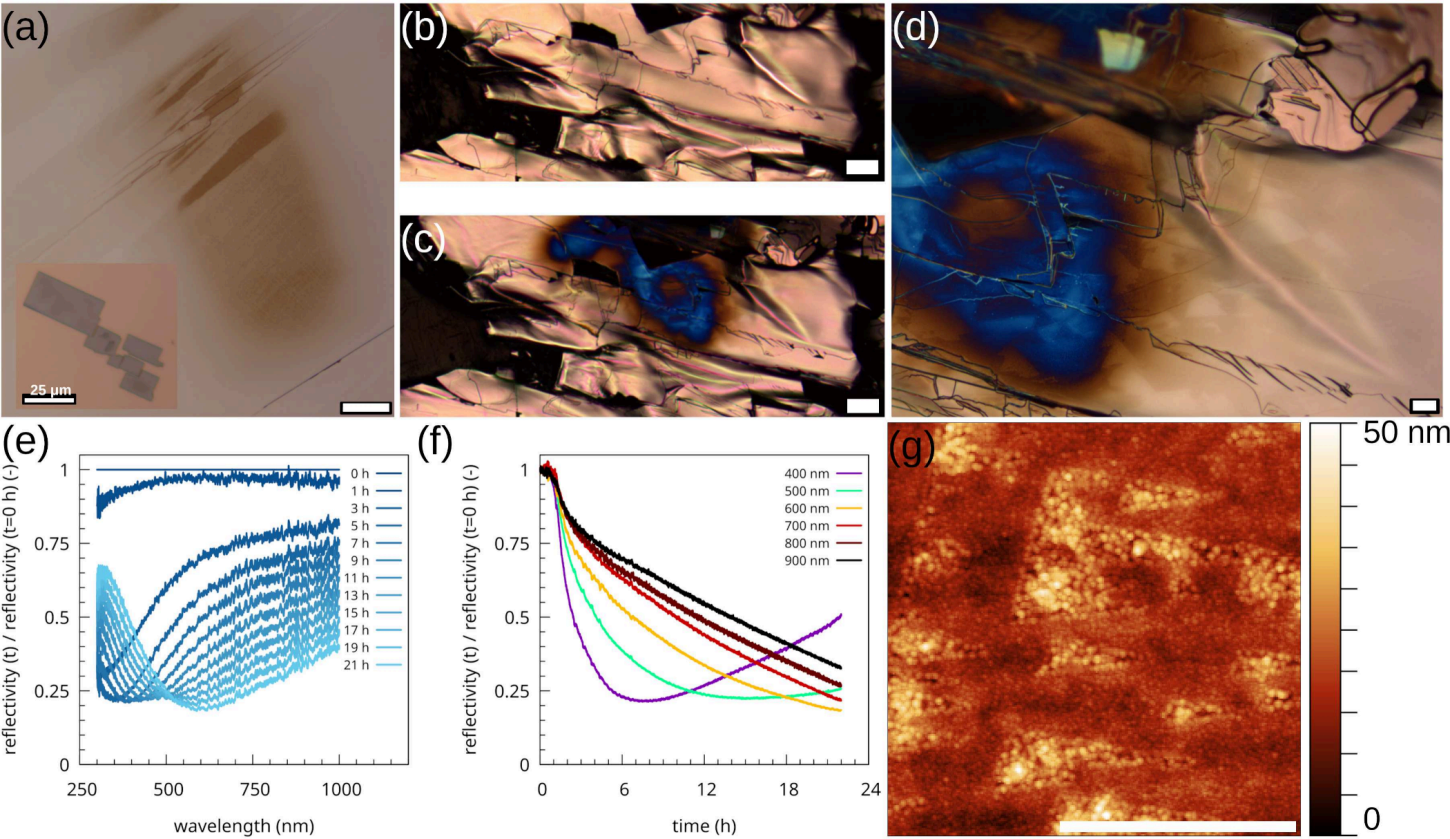
(a) Optical microscopy image of the surface of (PbS)_1.11_VS_2_ after illumination. Clearly visible are the brown elliptical area, where the light was focused, and variations in the intensity of the brown layer. The inset shows an exfoliated, approximately 100 nm thick, (PbS)_1.11_VS_2_ crystal, where nanoparticles were grown onto. Optical microscopy image of the area before (b) and after (c) illumination. (d) At the edge of the illuminated area, clearly triangular domains are visible. (e) Wavelength-dependent normalized reflectivity during illumination with white light. (f) Time-dependent normalized reflectivity for selected wavelengths. In both (e) and (f), the time of 0 h indicates a clean, freshly cleaved sample. (g) Topography measured using AFM of an illuminated triangular area, showing the presence of dense nanoparticle distribution on the moiré domains. Scale-bar in panel a is 50 μm, in panels b and c is 100 μm, in panel d is 25 μm, and in panel g is 4 μm.

When another crystal was illuminated at ambient conditions for almost 1 day, the shiny surface of the (PbS)_1.11_VS_2_ crystal changed within the light spot to a dark blue region, as visible in Figure 5b,c. When a higher magnification of the optical microscope is used, the characteristic triangular regions become visible, both in the blue area and in the brown area, as shown in Figure 5d. The optical reflectivity was measured during illumination of the sample, as shown in Figure 5b. In Figure 5e, the wavelength dependence of the normalized reflectivity of the whole inhomogeneous area is shown for different reaction times. Clearly visible is that already after 1 h, the total intensity drops. The intensity for the lower wavelengths decreases faster, which is visible as the slow change of color of the sample to various shades of brown. In the next hours of the reaction, the normalized reflectivity drops significantly for the lower wavelength range. However, the wavelength with the minimum normalized reflectivity slowly shifts to higher wavelengths, indicating the growth of the particles and their change in optical properties. This is clearly visible in Figure 5f, where the time dependence of the normalized reflectivity is shown for several selected wavelengths.

Finally, the topography of such light-induced growth domains, as presented in the AFM image in Figure 5g, shows that the density of particles is larger and more homogeneous for the illuminated samples compared to the naturally grown samples. It should be noted that, although the reaction seems to be photocatalytic, the light-induced reaction is more complex. UPS or UV–vis absorption spectroscopy suggests the crystals are metallic.^33^ Again, it can be expected that the presence of the moiré landscape, similar as for BiOCl,^16^ can spatially modify and thereby enhance the light absorption and/or dynamics of the photogenerated carriers.

To get an estimate of the reaction speed, the particle size was determined in the light-brown region, which has a diameter of approximately 15 nm after 22 h of growth, whereas blue particles reach a diameter up to 100 nm after 22 h of growth. Depending on the light intensity, the growth speed is on the order of a few nanometers per hour. Assuming the reacting alkane is C_20_H_42_ and a growth rate of 1 nm/hour, a production of 3 μmol/m^2^/hour is estimated. (PbS)_1.11_VS_2_ has a density of 5.1 g/cm^3^, so for crystals that are approximately 10 μm thick, the production is 0.6 μmol/g. It should be noted that the estimated reaction speed is for the diverse long hydrocarbon chains present in air, and the production could be higher for a well-defined reaction such as carbon dioxide or nitrogen reduction or hydrogen or oxygen evolution reaction.

As sliding ferroelectricity depends on the layer stacking, the influence of the layers closest to the surface is most important. The reactivity per unit mass can then be increased by exfoliation of the crystals. As shown for graphene, exfoliation to thin flakes is possible both manually and on a large scale. Thus, exfoliating the crystal to approximately 100 nm thick flakes (see inset of Figure 5a), the reactivity per unit mass can be increased by a factor of a hundred. Similarly, liquid exfoliation and the subsequent deposition of the sliding ferroelectric catalyst onto a substrate should allow the production of the particles to be scaled up.

Furthermore, the dense placement of the particles suggests that the reaction happens homogeneously at the whole surface and is not limited by defects or edges, as happens in 2H-stacked TMD catalysts. Even though the catalytic properties of VS_2_ are comparable to MoS_2_,^53,54^ the presence of the TMD VS_2_ is not enough to explain the domain-dependent catalytic reactivity.

The fast growth of the particles, combined with the presence of regions with a different color, can also be used as a moiré ferroelectric decoration technique. The various domains with different colors can hint at the different stacking and twisting orders present in the crystal, which all have their own characteristic catalytic activity.

Finally, to identify if the growth of carbon nanoparticles on (PbS)_1.11_VS_2_ is a general property of 2D crystals or the presence of moiré domains is a prerequisite, the accelerated growth technique is used to try to grow particles onto a MoS_2_-crystal and onto a misfit layer compound (BiS)_1.24_CrS_2_-crystal. The misfit layer compound (BiS)_1.24_CrS_2_ forms similar as (PbS)_1.11_VS_2_ twins, but no reconstruction into triangular moiré domains could be detected, as visible in Figure 6b.^55^ If one looks very carefully at the bottom-edge of the highest terrace (most yellow area) in Figure 6b, it can be seen that the edge is not straight, but the particles removed a small part of the flake. For both materials, only a small fraction of particles are formed on edges and defects, the typical catalytic sites in transition metal dichalcogenides systems.^56^ In clear contrast, the catalytic reaction on the (PbS)_1.11_VS_2_ surface happens at the whole basal plane of the moiré domains with the right polarization, and no removal of the edges of flakes was detected, highlighting the stability of the system.

**Figure 6:**
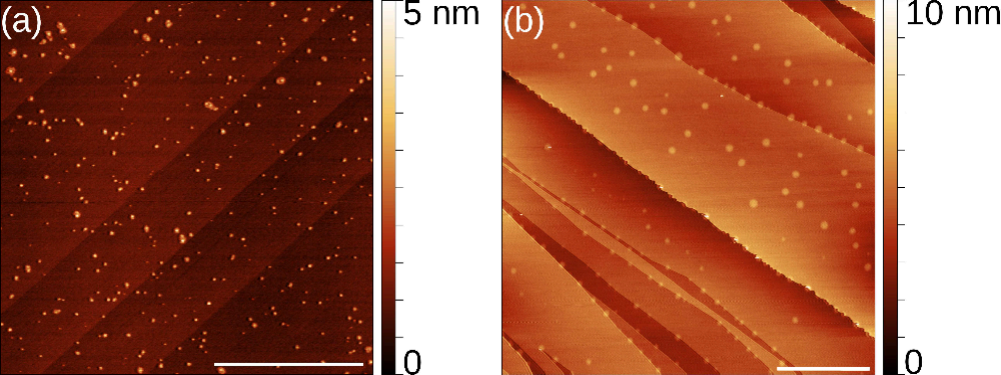
AFM topography images of (a) MoS_2_ and (b) (BiS)_1.24_CrS_2_ surfaces after an attempt to grow carbon nanoparticles by using the accelerated growth method. Scale bar in panel a is 2 μm and in panel b is 5 μm.

## Conclusion

To conclude, we have shown the catalytic properties of sliding ferroelectric domains in 2D misfit layer compound crystals. The presence of micrometer domains allows us to straightforwardly follow the selective growth of carbon on only one polarization domain orientation of the moiré sliding ferroelectric under ambient, room-temperature conditions, without the need of external sources such as heat or illumination. The particle growth speed can be significantly accelerated by using white light illumination.

The presented work highlights the potential of 2D materials in catalysis and surface chemistry control. The possibility to use the properties of the moiré landscapes present in 2D materials stacked with a small twist angle will open a fascinating new direction to control surface chemistry.

## Supplementary Material



## Notes

Additional optical microscope image of Figure 1c after 1 year, full Raman spectra of Figure 3b, and optical reflectivity of bulk (PbS)_1.11_VS_2_ crystal (PDF)
