# Supplementary material for: Sliding Ferroelectric Catalyst for Carbon Nanoparticle Generation
Source: ACS Appl Nano Mater. 2025 Jun 2;8(23):11847–55. doi: 10.1021/acsanm.5c01032 (PMC12172014; doi:10.1021/acsanm.5c01032)
Supplement: Supplementary file 1 [file an5c01032_si_001.pdf]

Supporting Information
Sliding Ferroelectric Catalyst for Carbon Nanoparticle Generation

Johana Vaníčková,[†] Klára Uhlířová,[‡] Jiří Volný,[‡] and Tim Verhagen[*,†,‡]

†*Institute of Physics, Czech Academy of Sciences, Na Slovance 2, Prague 8, 182 00, Czech Republic*

‡*Faculty of Mathematics and Physics, Charles University, Ke Karlovu 3, 121 16, Prague 2, Czech Republic*

E-mail: verhagen@fzu.cz

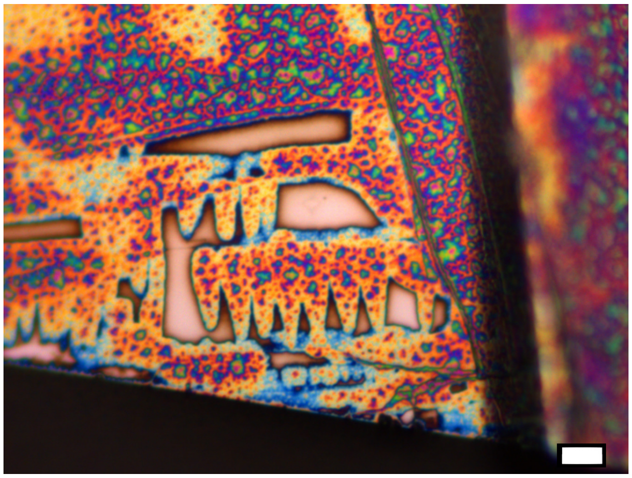

Figure S1: Optical microscope image of Fig. 1(f) after 1 year. The scale-bar is 25 μm.

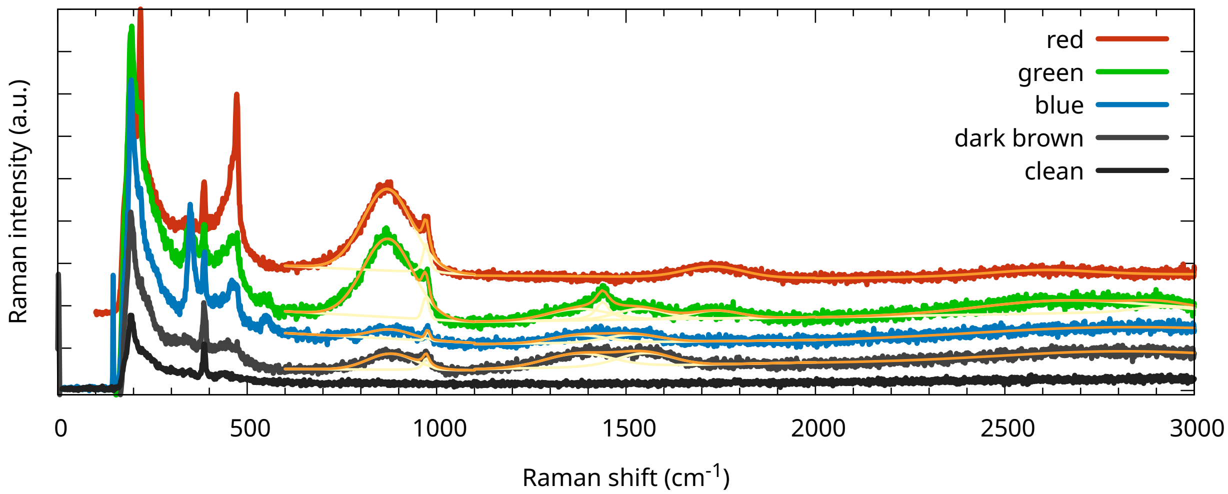

Figure S2: Raman spectra of the clean $(PbS)_{1.11}VS_2$ crystal and of the $(PbS)_{1.11}VS_2$ crystal with a carbon nanoparticles with a different size, characterised by their colour. The fitted Raman bands are shown using the yellow colour and the sum the fitted bands in the D, G and 2D region are shown using the orange colour. Each spectrum is vertically offset for clarity.

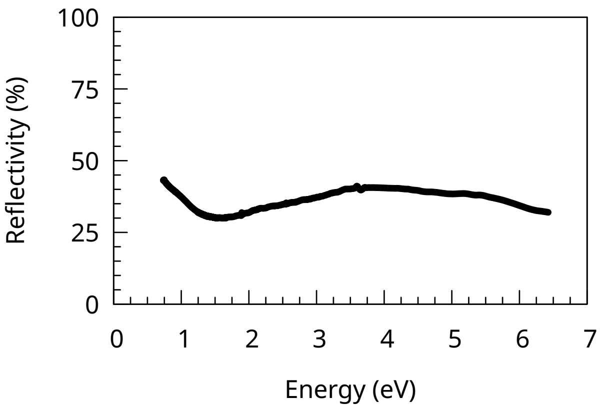

Figure S3: Reflectivity of a bulk $(PbS)_{1.11}VS_2$ crystal.
